# Single‐Cell Transcriptomics and Integrated Bioinformatic Analysis Reveal Critical Biomarkers and Immune Infiltration Characteristics in Osteoarthritis

**DOI:** 10.1155/genr/1174568

**Published:** 2026-01-06

**Authors:** Tiantian Gao, Chongshan Yang, Yikang Bi, Pingzhou Zou, Ma Wan, Shenghui Lan, Yuan Song, Yafeng Xu

**Affiliations:** ^1^ Department of Orthopaedics, The Eighth People’s Hospital, Jiangsu University, Shanghai, 200235, China, ujs.edu.cn; ^2^ Department of Orthopaedics, Xuhui Branch of The Sixth People’s Hospital, Shanghai Jiao Tong University, Shanghai, 200233, China, sjtu.edu.cn; ^3^ Department of Orthopaedics, Suzhou Wujiang District Hospital of Traditional Chinese Medicine (Suzhou Wujiang District Second People’s Hospital), Suzhou, 215000, Jiangsu, China

**Keywords:** AVPR1A, BMP1, chondrocyte heterogeneity, molecular docking, NR4A2, osteoarthritis, single-cell RNA sequencing

## Abstract

**Background:**

Osteoarthritis (OA) is a complex, progressive joint disease characterized by cartilage degradation and inflammation. Traditional bulk tissue analyses have limited our understanding of the cellular diversity within OA tissues.

**Methods:**

This study employed scRNA‐seq and integrated bioinformatic analyses to investigate the cellular composition and molecular pathways involved in OA. Publicly available datasets were analyzed to identify differentially expressed genes (DEGs) and enriched pathways. The genes, such as *NR4A2*, *BMP1*, and *AVPR1A*, were selected for further analysis. Molecular docking studies were conducted to explore the interaction with two identified compounds. Additionally, immune infiltration characteristics were analyzed using gene set variation analysis (GSVA) and correlation with key OA‐associated genes.

**Results:**

We analyzed cartilage samples from OA and normal individuals (GSE220243) and identified eight distinct chondrocyte subpopulations, with significant pathway enrichment in TNF, TGF‐β, and PI3K–Akt signaling pathways. Further differential gene expression analysis of GSE114007 identified 2247 genes, including 26 key OA‐associated drug targets, such as *NR4A2*, *BMP1*, and *AVPR1A*, which demonstrated strong diagnostic potential (AUC > 0.70) across multiple cohorts. Immune infiltration analysis revealed significant correlations between these key genes and immune cell subsets, highlighting their roles in the inflammatory microenvironment of OA. Additionally, molecular docking studies suggested that bexarotene has a favorable binding affinity for NR4A2, BMP1, and AVPR1A, making it a promising therapeutic candidate.

**Conclusion:**

Our findings provide new insights into the molecular landscape of OA, offering valuable biomarkers and therapeutic targets for future OA interventions.

## 1. Introduction

Osteoarthritis (OA) is a complex and multifactorial joint disease, characterized by the progressive degeneration of articular cartilage, synovial inflammation, and alterations in subchondral bone [1]. Despite its widespread occurrence, the molecular mechanisms driving OA progression remain incompletely understood, and effective disease‐modifying treatments are still lacking. Traditional bulk tissue analyses have provided valuable insights into the molecular landscape of OA; however, they fall short in capturing the cellular heterogeneity and complex interactions within the joint microenvironment.

Recent advances in single‐cell transcriptomics have revolutionized our understanding of cellular diversity and gene expression dynamics at an unprecedented resolution [2]. By enabling the dissection of individual cell populations within complex tissues, this approach has unveiled previously unrecognized cellular subtypes and states that contribute to disease pathogenesis [3, 4]. In the context of OA, single‐cell technologies offer the potential to identify critical biomarkers and therapeutic targets by elucidating the specific roles of different cell types, including chondrocytes, synoviocytes, and immune cells, in disease progression [5, 6].

Immune infiltration, a hallmark of OA, plays a pivotal role in modulating the inflammatory milieu of the joint [7]. Macrophages, T cells, and other immune cells infiltrate the synovium and contribute to the chronic inflammation and tissue destruction characteristic of OA [8, 9]. Understanding the cellular and molecular mechanisms underlying immune infiltration is essential for developing targeted therapies aimed at mitigating inflammation and halting disease progression.

In this study, we employed single‐cell transcriptomics combined with integrated bioinformatic analysis to unravel the cellular heterogeneity and immune infiltration characteristics in OA. By analyzing the transcriptomes of individual cells from OA‐affected joints, we aimed to identify critical biomarkers and potential therapeutic targets that could lead to more precise and effective treatment strategies. Furthermore, we explored the immune landscape of OA, providing insights into the complex interactions between immune cells and resident joint cells that drive inflammation and tissue damage.

Our findings underscore the importance of leveraging single‐cell technologies to decode the intricate molecular networks involved in OA and pave the way for novel therapeutic approaches that address the disease at its cellular roots. This study not only advances our understanding of OA pathogenesis but also highlights the potential of single‐cell transcriptomics as a powerful tool in the search for disease biomarkers and therapeutic targets.

## 2. Methods

### 2.1. Data Collection and Preparation

Publicly available gene expression data related to OA were sourced from the NCBI Gene Expression Omnibus (GEO) database [10]. A comprehensive search was conducted using the keyword “osteoarthritis” with the filter set to “Homo sapiens” to identify datasets pertinent to the study of OA in human tissues. We selected multiple datasets (GSE220243, GSE82107, GSE98918, and GSE114007) that provided robust coverage of synovial joint, meniscus, and knee cartilage tissues, essential for understanding OA pathology. A summary of the datasets used in the study is provided in Table 1. These datasets encompass various experimental platforms, including high‐throughput sequencing and expression profiling by array, allowing for a comprehensive analysis of gene expression changes across different tissue types affected by OA.

**Table 1:** Detailed information of datasets.

GEO ID	GPL ID	Species	OA samples (*n*)	Control samples (*n*)	Organization source	Experiment type	References
GSE220243	GPL18573	*Homo sapiens*	6	6	Knee cartilage tissues	Expression profiling by high‐throughput sequencing	PMID: 36564153
GSE82107	GPL570	*Homo sapiens*	10	7	Synovial tissues	Expression profiling by array	PMID: 27870898
GSE98918	GPL20844	*Homo sapiens*	12	12	Meniscus tissues	Expression profiling by array	PMID: 29258882
GSE114007	GPL11154 and GPL18573	*Homo sapiens*	20	18	Knee cartilage tissues	Expression profiling by high‐throughput sequencing	PMID: 30081074

Furthermore, we identified OA‐related drugs and their corresponding target genes using the ChEMBL database (https://www.ebi.ac.uk/chembl/) [11]. In particular, we focused on compounds categorized as bone metabolism regulators and glucocorticoid hormones, as these drug classes have been previously reported to be effective in the treatment of OA. The relevant data were filtered based on drug efficacy, known therapeutic targets, and relevance to bone and cartilage metabolism.

### 2.2. Quality Control (QC) and Data Preprocessing

To ensure the robustness and reliability of the single‐cell RNA sequencing data, we performed stringent QC measures using the Seurat package. Cells with low quality were filtered out by applying the following criteria: Each gene was required to be expressed in at least three cells, and each cell was required to express a minimum of 50 genes. This filtering step was essential to remove cells with insufficient RNA content or technical artifacts that could bias downstream analyses.

To further refine the dataset, we calculated the proportion of mitochondrial genes and ribosomal RNA (rRNA) within each cell using the PercentageFeatureSet function in Seurat. High mitochondrial or rRNA content often indicates cellular stress or low‐quality cells, which can compromise data integrity. Cells exhibiting abnormally high percentages of mitochondrial or rRNA content were excluded from further analysis.

Next, we identified highly variable genes across the dataset using the FindVariableFeatures function, which captures the most informative genes that contribute to cellular heterogeneity. These variable genes were then used in the subsequent steps of data normalization and dimensionality reduction. Data normalization was performed using the ScaleData function in Seurat, which centers and scales the expression values for each gene across all cells, thereby minimizing technical variations and facilitating the comparison of gene expression across cells. Principal component analysis (PCA) was then employed for dimensionality reduction, allowing the identification of key principal components that capture the majority of the variance in the data.

### 2.3. Cell Annotation and Clustering Analysis

To classify and annotate cell types within the single‐cell RNA sequencing dataset, we first performed clustering analysis using the Seurat package. The clustering process involved constructing a shared nearest neighbor (SNN) graph using the FindNeighbors function, with dimensionality (dims) set to 15 to capture the most informative principal components. We then applied the FindClusters function with a resolution parameter of 0.2, which allowed for the identification of discrete cell populations. This analysis resulted in the identification of seven distinct clusters, each representing a unique cell population. Following clustering, cell‐type annotation was conducted using the SingleR package, which leverages reference transcriptomic datasets to assign cell identities based on gene expression profiles. This approach enabled the accurate annotation of the seven clusters into two major cell types, providing a clear understanding of the cellular composition within the dataset.

To further characterize the clusters, differential expression analysis was performed using the FindAllMarkers function in Seurat. This function identified marker genes that were significantly upregulated within each cluster, serving as unique molecular signatures for the respective cell populations. Subsequently, the identified marker genes for each cluster were subjected to enrichment analysis using the clusterProfiler package. This analysis involved determining the biological processes (BPs), molecular functions (MFs), and pathways that were overrepresented among the marker genes, thereby providing insights into the functional roles of the distinct cell populations identified in the dataset.

### 2.4. Differential Gene Expression Analysis

To identify differentially expressed genes (DEGs) associated with OA, we utilized the GSE114007 dataset. The dataset was first normalized using the normalizeBetweenArrays function from the limma package, ensuring that the expression data were comparable across samples. Samples were then divided into OA and normal control groups for differential expression analysis.

Differential expression analysis was performed using the limma package, with the significance threshold set to an adjusted *p*‐value (adj.p) < 0.05 and an absolute log fold change (|logFC|) > 1. To visualize these results, a volcano plot was generated using the ggplot2 package, highlighting the most significantly altered genes. Additionally, a heat map was created using the pheatmap package to display the expression patterns of the DEGs across all samples, providing insights into the overall transcriptional changes associated with OA.

### 2.5. Identification of OA‐Associated Genes

To explore potential therapeutic targets, we obtained a list of 205 OA‐related drug target genes from the ChEMBL database. These genes were intersected with the DEGs identified from the GSE114007 dataset, resulting in the identification of 26 key OA‐associated genes. A Venn diagram was generated using the venn package to illustrate the overlap between the DEGs and the drug target genes, highlighting these 26 key genes as potential therapeutic targets in OA.

### 2.6. Gene Set Variation Analysis (GSVA) and Correlation Analysis

To further investigate the functional relevance of the identified key genes in specific cell populations, we conducted GSVA on marker genes from different single‐cell clusters within the normalized GSE114007 dataset. GSVA was performed using the GSVA package, focusing particularly on Fibroblasts_6, a cluster identified as having significant relevance to OA pathophysiology. The results were visualized with boxplots generated using the ggplot2 package, comparing GSVA scores between the OA and normal groups. Statistical significance was assessed using the Wilcoxon test (wilcox.test), with *p*‐values calculated to determine the significance of differences observed. Subsequently, Pearson’s correlation analysis was conducted to explore the relationships between GSVA scores of the seven single‐cell clusters and the expression levels of the 26 key OA‐associated genes. The correlation coefficients were visualized using a heat map generated by the pheatmap package, providing an overview of the associations between key genes and cellular processes across different cell populations.

### 2.7. Validation of Key Genes

To validate the diagnostic potential of the 26 key OA‐associated genes, we extracted their expression profiles and corresponding sample group information from six independent datasets: GSE82107, GSE98918, and GSE114007. Diagnostic models were constructed for each gene using the pROC package in R to generate receiver operating characteristic (ROC) curves, with OA vs. normal control as the comparison groups. The area under the curve (AUC) was calculated for each ROC curve, with an AUC value greater than 0.7 considered indicative of significant diagnostic value. For a more comprehensive analysis, we also constructed a logistic regression model incorporating multiple genes using the RMS package in R. This multivariate model was also evaluated using ROC curves to assess the combined diagnostic power of the key genes. The AUC values from these models provided a robust measure of the predictive accuracy of the key genes for distinguishing OA from normal samples.

### 2.8. Transcriptomic Enrichment Analysis

The DEGs from the GSE114007 dataset were subjected to Gene Ontology (GO) and Kyoto Encyclopedia of Genes and Genomes (KEGG) pathway enrichment analyses using the clusterProfiler package in R. Briefly, the DEGs were input into clusterProfiler, and significant terms (p.adjust < 0.05) were extracted for subsequent annotation. GO terms were grouped into BP, cellular component (CC), and MF. For KEGG pathways, significant pathways were likewise identified based on the threshold of p.adjust < 0.05. The enrichplot package was then employed to generate dot plots illustrating the top 10 most significantly enriched terms in each of the GO categories (BP, CC, and MF) and in KEGG pathways. These analyses provided comprehensive insights into the functional and BPs relevant to OA pathogenesis.

### 2.9. Immune Infiltration Analysis

To explore the relationship between immune cell infiltration and the 12 key OA‐associated genes, we performed single‐sample gene set enrichment analysis (ssGSEA) using the GSVA package in R. The analysis focused on gene sets associated with 28 different immune cell types, using the GSE55235 dataset. The resulting GSVA scores for each immune cell type were then correlated with the expression levels of the 12 key genes using Pearson’s correlation analysis. A heat map was generated using the pheatmap package to visualize these correlations, with statistical significance indicated ^∗∗∗^ *p* < 0.001, ^∗∗^ *p* < 0.01, ^∗^ *p* < 0.05, and ns (not significant). The color gradient from blue to red reflects the correlation coefficient (cor), indicating the strength and direction of the correlation.

To further characterize the tumor microenvironment in OA, we calculated the ESTIMATEScore, StromalScore, and ImmuneScore for the GSE114007 dataset using the estimate package in R. These scores provide quantitative measures of the stromal and immune cell content within the samples, as well as an estimate of tumor purity. The scores were then correlated with the expression levels of the 26 key OA‐associated genes using Pearson’s correlation analysis. The results were visualized in a heat map, similar to the immune infiltration analysis, with significance and correlation strength indicated by the same color and symbol scheme.

### 2.10. Molecular Docking

Compounds putatively targeting the diagnostic genes *AVPR1A*, *BMP1*, and *NR4A2* were retrieved from the ChEMBL database. From these, two candidate small‐molecule ligands were selected—bexarotene (DB00307) and meclofenamic acid (DB00939)—based on their putative relevance to OA pathophysiology. Protein structures of AVPR1A, BMP1, and NR4A2 were downloaded in Protein Data Bank (PDB) format from the PDB, whereas the corresponding ligands were obtained in MOL format from PubChem.

Docking studies were performed using the AutoDock Tools suite (version 1.5.7). Briefly, protein structures were prepared by removing water molecules, adding polar hydrogens, and assigning Gasteiger partial charges. Ligand structures were similarly prepared by energy minimization and conversion to the required PDBQT format. A grid box was configured around the predicted active site of each target protein to ensure comprehensive sampling of potential ligand binding pockets. The Lamarckian genetic algorithm was applied for docking, with default parameter settings unless otherwise specified. The most favorable binding modes were selected based on the lowest predicted binding energy and a cluster analysis of docking poses. Key interacting residues were identified by analyzing hydrogen bonds, hydrophobic contacts, and electrostatic interactions in the final docked complexes.

### 2.11. Construction of Drug–Target–Pathway Interaction Networks

The drug–target–pathway interaction network was constructed to visualize the relationships among the identified drugs, hub genes, and enriched pathways [12]. The network was designed to illustrate how these components interact with each other, providing insights into the potential therapeutic mechanisms. Only the top 20 pathways with the lowest *p*‐value were selected for GO, and only the top 20 pathways with the lowest *p*‐value were selected for KEGG. Cytoscape, a powerful tool for network analysis and visualization, was employed to create the interaction network.

### 2.12. Quantitative Real‐Time PCR (qRT‐PCR) Validation of Hub Genes

Cartilage tissue samples were obtained from OA patients undergoing total knee arthroplasty and from non‐OA donors who served as healthy controls. All procedures involving human tissues were approved by the Ethics Committee of Shanghai Eighth People’s Hospital (Approval Number: 2025‐124‐25). Written informed consent was obtained from all participants before sample collection.

Total RNA was extracted and reverse‐transcribed into first‐strand cDNA using a commercial cDNA Synthesis Kit. Subsequently, real‐time PCR was conducted on the CFX96 Real‐Time PCR System (Bio‐Rad, Hercules, CA, USA) under the following cycling protocol: initial denaturation at 95°C for 1 min, followed by 40 cycles of denaturation (95°C, 20 s), annealing (55°C, 20 s), and extension (72°C, 30 s). Primer sequences—designed with the NCBI Primer BLAST tool—are detailed in Supporting Table S3.

## 3. Results

### 3.1. Single‐Cell RNA Sequencing Reveals Distinct Cellular Subpopulations in Knee Cartilage Tissues

To decipher the cellular heterogeneity of knee cartilage in OA and non‐OA (normal) individuals, we performed single‐cell RNA sequencing (scRNA‐seq) on cartilage samples (GSE220243). After stringent QC (Figure S1), cells expressing fewer than 50 genes or genes detected in fewer than three cells were excluded. Mitochondrial and ribosomal gene contents were calculated using the PercentageFeatureSet function in Seurat. A total of 11 samples passed QC, yielding a high‐quality dataset of transcriptomes. Highly variable genes were identified with the FindVariableFeatures function, and data were normalized, scaled, and subjected to PCA for dimensionality reduction.

Subsequent clustering was performed with the FindNeighbors and FindClusters functions (dimensions = 15 and resolution = 0.2), revealing seven major clusters and an additional minor cell population. Cell‐type annotation was assisted by SingleR, which classified the predominant clusters as chondrocytes (Figure 1(a)). We further subdivided these chondrocyte clusters based on transcriptional signatures, yielding eight subpopulations (Chondrocytes_1 through Chondrocytes_8). Notably, both normal and OA groups contributed to each subpopulation, albeit at different proportions (Figures 1(b), 1(c), 1(d)). Marker gene analyses (FindAllMarkers function) confirmed distinct transcriptional profiles for each chondrocyte subpopulation, illustrated by the subpopulation‐specific expression patterns of representative marker genes (Figure 1(c)).

Figure 1Single‐cell transcriptomic landscape of knee cartilage chondrocytes. (a) t‐SNE plot displaying the eight chondrocyte subpopulations (Chondrocytes_1–8) identified through unsupervised clustering. Each color indicates a distinct subpopulation. (b) t‐SNE plot highlighting the distribution of OA (red) and normal (green) cells, illustrating the overlap yet distinct proportions of diseased and healthy cells across chondrocyte subpopulations. (c) Dot plot representing the average expression and percentage of cells expressing representative marker genes within each chondrocyte subpopulation. Circle size indicates the percentage of cells expressing a given gene; color intensity denotes the average expression level. (d) Bar plots showing (left) the proportion of normal (orange) and OA (blue) cells in each chondrocyte subpopulation and (right) the corresponding cell numbers, revealing differences in subpopulation abundance between the two groups.

**Figure:**
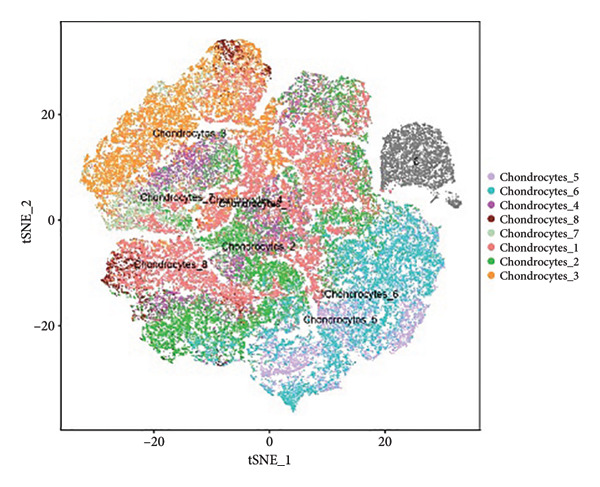
(a)

**Figure:**
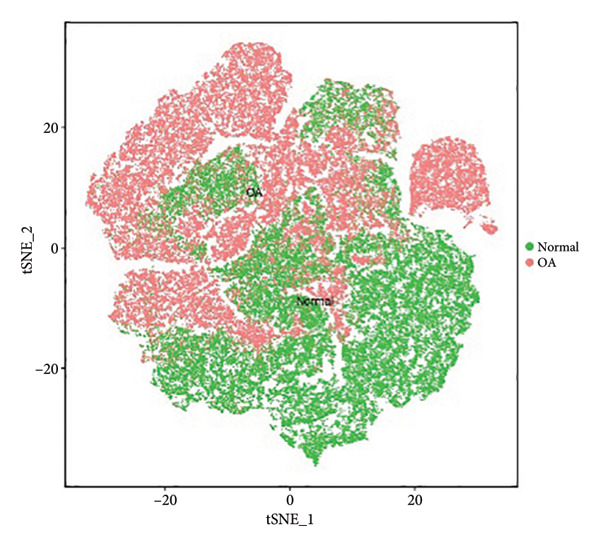
(b)

**Figure:**
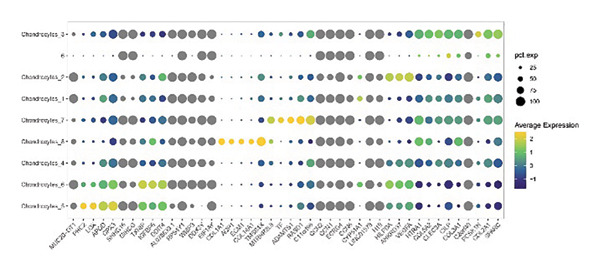
(c)

**Figure:**
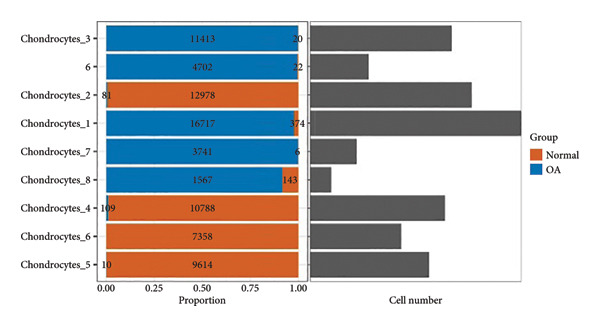
(d)

**Figure 2:**
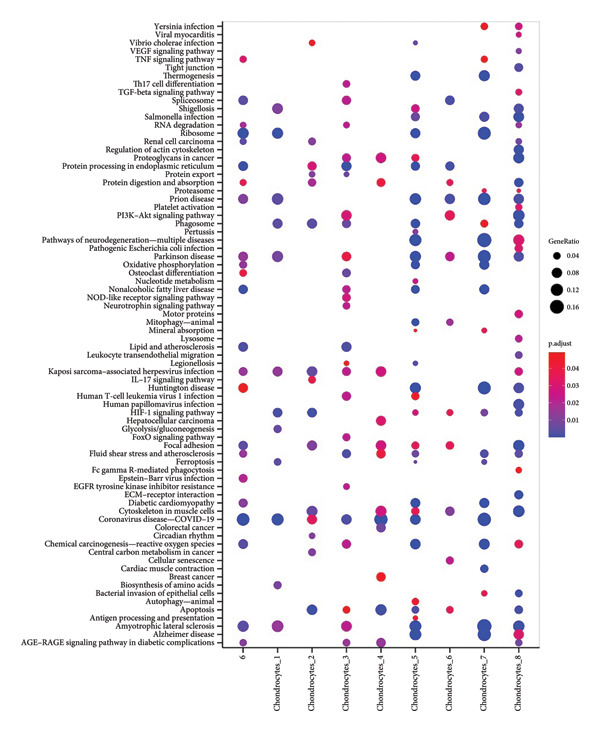
KEGG pathway enrichment of distinct chondrocyte subpopulations. Bubble plot displaying enriched KEGG pathways for seven of the chondrocyte subpopulations (Chondrocytes 1–3 and 5–8) identified in scRNA‐seq analysis. The size of each bubble indicates the gene ratio (proportion of genes within a pathway that were differentially expressed in that subpopulation), and the color scale depicts the adjusted *p*‐value (p.adjust). Pathways such as “TNF signaling,” “TGF‐β signaling,” and “PI3K–Akt signaling” were significantly enriched in multiple subpopulations, whereas “Fibroblasts_4” did not yield significant enrichment.

We next performed enrichment analysis for each subpopulation using the ClusterProfiler package. KEGG pathway overrepresentation (Figure 2) revealed that seven of the chondrocyte subpopulations displayed significant functional enrichment in pathways including “TNF signaling,” “TGF‐β signaling,” and “PI3K‐Akt signaling.” In contrast, one fibroblast‐enriched cluster (“Fibroblasts_4”) did not exhibit significant enrichment under our statistical thresholds. These results underscore the functional diversity among chondrocyte subpopulations, highlighting specific signaling cascades potentially implicated in OA pathogenesis.

Collectively, our single‐cell transcriptomic analysis uncovers eight transcriptionally distinct subpopulations within knee cartilage, with marked differences in subpopulation compositions and gene expression programs between normal and OA tissues. This work provides key insights into the cellular landscape of OA‐affected cartilage and sheds light on potential pathways that underlie disease progression.

### 3.2. Identification of Key DEGs in OA

To elucidate the transcriptional alterations underpinning OA, we reanalyzed the GSE114007 dataset after normalization with the normalizeBetweenArrays function in the limma package. A total of 2247 DEGs were detected, including 1311 upregulated and 936 downregulated genes. Visual inspection via volcano plotting (Figure 3(a)) confirmed marked expression shifts in several genes, such as *COL1A1*, *POSTN*, and *IL11*, which stood out among the top upregulated genes in OA cartilage, and *HILPDA*, *ADM*, and *ATF3*, which were the top downregulated genes in OA cartilage. This heat map underscored distinct transcriptional signatures between OA and control samples, clustering OA tissues separately from normal tissues and suggesting a profound molecular divergence in diseased cartilage (Figure 3(b)).

Figure 3Identification of key differentially expressed genes in osteoarthritis. (a) Volcano plot showing differentially expressed genes in OA versus normal (control) cartilage (|log_2FC| > 1, adjusted *p* < 0.05). Red dots represent significantly upregulated genes, blue dots represent significantly downregulated genes, and black dots represent nonsignificant genes. Key genes are labeled for emphasis. (b) Heat map of the 2247 DEGs in OA (pink bar) and normal (green bar) samples. Each row represents a single DEG, and the color scale indicates relative expression levels (red, upregulated; blue, downregulated). (c) Venn diagram illustrating the intersection of 205 putative OA drug–target genes from ChEMBL with the 2247 DEGs, yielding 26 key OA‐associated genes. (d) Boxplot of GSVA scores derived from the marker genes of the Chondrocytes_1 subpopulation (single‐cell data) applied to the GSE114007 dataset. Wilcoxon’s rank‐sum test indicates a significant difference in GSVA scores between OA and normal samples (*p* = 0.0023). (e) Pearson’s correlation heat map of the GSVA scores (columns) in nine chondrocyte subpopulations versus the 26 key OA‐associated genes (rows). The color scale represents the correlation coefficient (positive in red and negative in blue), with asterisks denoting significance (^∗^ *p* < 0.05, ^∗∗^ *p* < 0.01).

**Figure:**
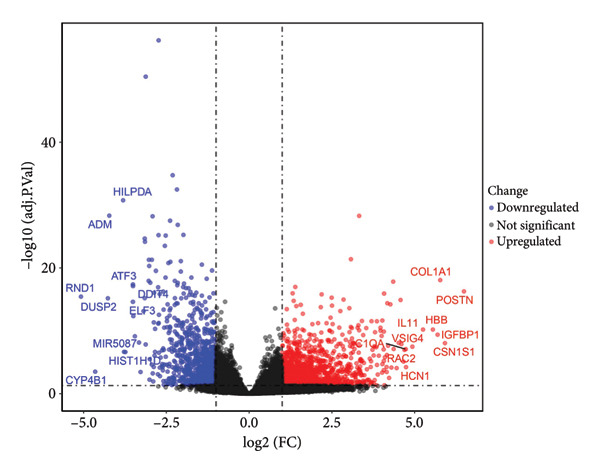
(a)

**Figure:**
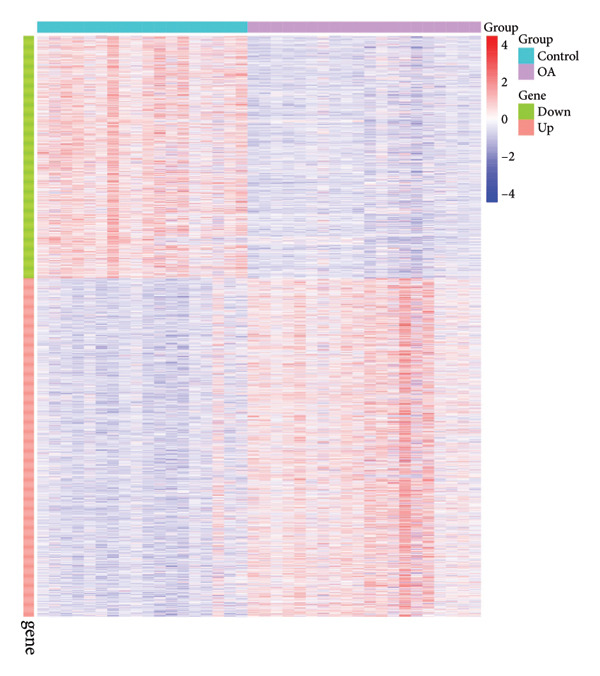
(b)

**Figure:**
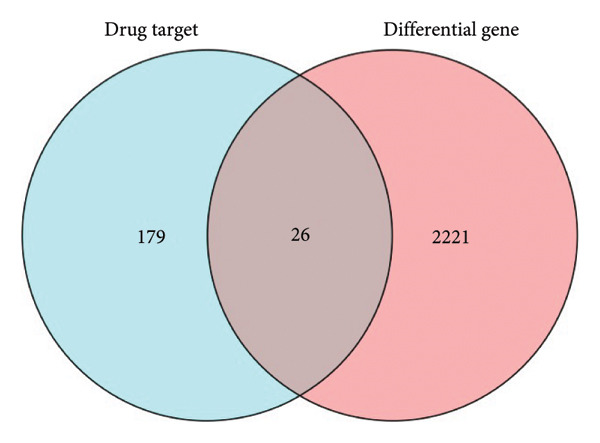
(c)

**Figure:**
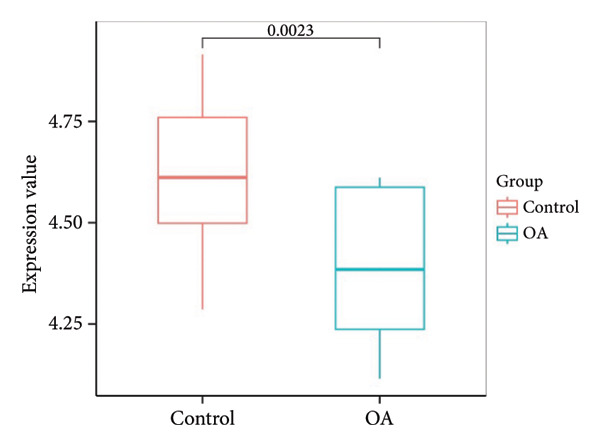
(d)

**Figure:**
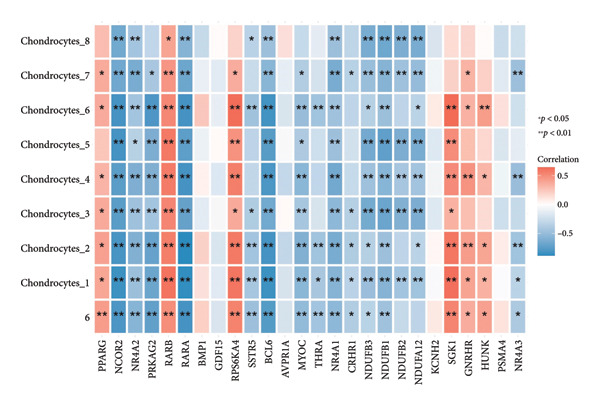
(e)

Given the therapeutic implications of DEGs, we next collated 205 putative OA therapeutic target genes from the ChEMBL database and intersected them with our DEG set, yielding 26 genes designated as key OA‐associated drug targets (Figure 3(c)), including *PPARG*, *NCOR2*, *NR4A2*, *PRKAG2*, *RARB*, *RARA*, *BMP1*, *GDF15*, *RPS6KA4*, *SSTR5*, *BCL6*, *AVPR1A*, *MYOC*, *THRA*, NR4A1, CRHR1, *NDUFB3*, *NDUFB1*, *NDUFB2*, *NDUFA12*, *KCNH2*, *SGK1*, *GNRHR*, *HUNK*, *PSMA4*, and *NR4A3*. We then conducted GSVA on marker genes identified from the single‐cell clusters in our parallel scRNA‐seq dataset (specifically GSE220243) using the normalized GSE114007 data as a reference. Boxplot analysis of GSVA scores for the Chondrocytes_1 subpopulation demonstrated a significant reduction in OA relative to normal tissues (*p* = 0.0023; Figure 3(d)), indicating that this cluster’s transcriptional signature is profoundly altered in OA.

Finally, we assessed how the 26 key DEGs correlated with GSVA scores across all nine chondrocyte subpopulations (Chondrocytes_1–8 and an additional minor cluster). Pearson’s correlation analysis revealed significant positive and negative associations (*p* < 0.05 or *p* < 0.01) between specific subpopulation GSVA scores and the 26 candidate drug target genes, as displayed in the correlation heat map (Figure 3(e)). These findings highlight putative molecular drivers and potential therapeutic targets in OA pathogenesis, warranting further functional validation.

### 3.3. The Validations of Key DEGs

To assess the diagnostic utility of the 26 key OA‐associated genes identified in our integrated analysis, we examined their expression profiles and corresponding clinical classifications (OA versus normal) in three independent validation cohorts (GSE114007, GSE82107, and GSE98918). Among the 26 candidate genes, *NR4A2*, *BMP1*, and *AVPR1A* consistently exhibited robust discriminatory power (AUC > 0.70) across all three datasets, underscoring their potential as reliable biomarkers for OA diagnosis. The remaining genes displayed variable performance, with some reaching diagnostic significance only in specific cohorts (Table S1). Taken together, these results highlight *NR4A2*, *BMP1*, and *AVPR1A* as strong candidates warranting further functional and translational investigation in the context of OA as seen in Figure 4.

Figure 4ROC analysis of key candidate genes across the three datasets. (a–c) ROC curves for *NR4A2* in GSE114007, GSE82107, and GSE98918, respectively, demonstrating high diagnostic potential (AUC range: 0.786–0.951); (d–f) ROC curves for *BMP1* in GSE114007, GSE82107, and GSE98918, respectively (AUC range: 0.843–0.958); (g–i) ROC curves for *AVPR1A* in GSE114007, GSE82107, and GSE98918, respectively (AUC range: 0.701–0.847).

**Figure:**
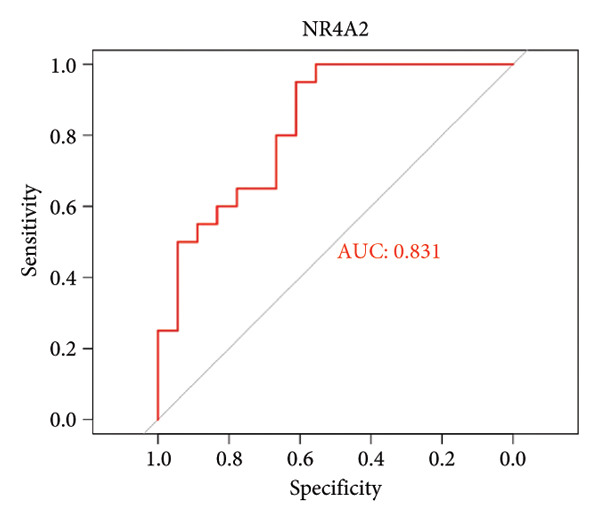
(a)

**Figure:**
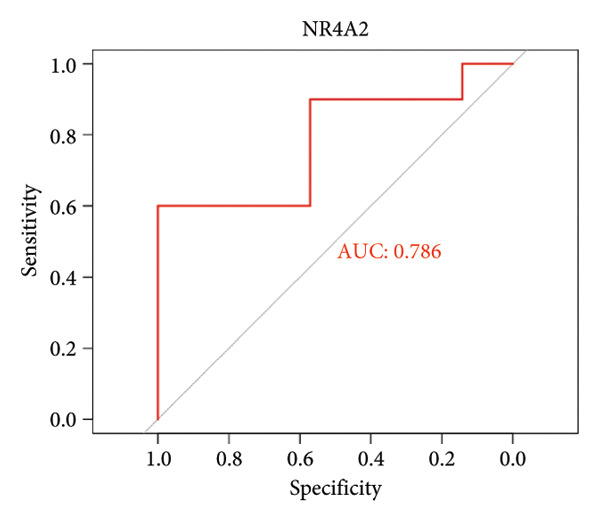
(b)

**Figure:**
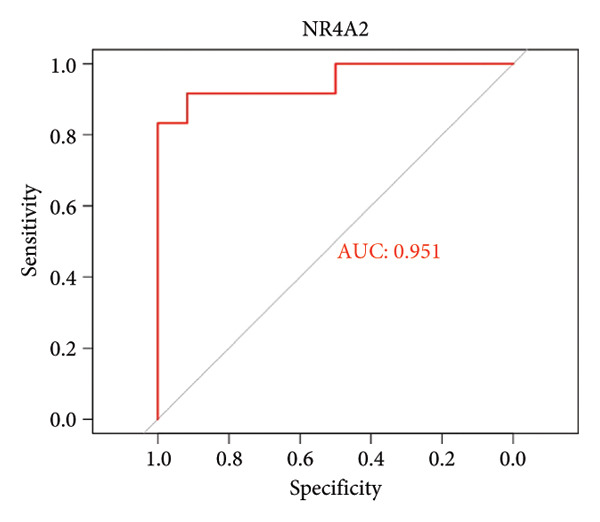
(c)

**Figure:**
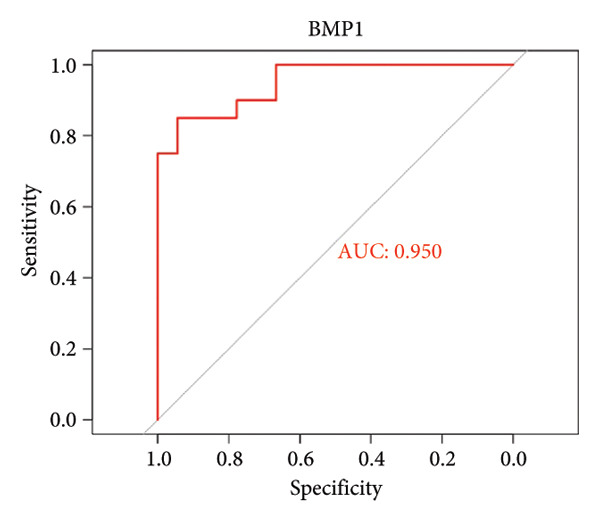
(d)

**Figure:**
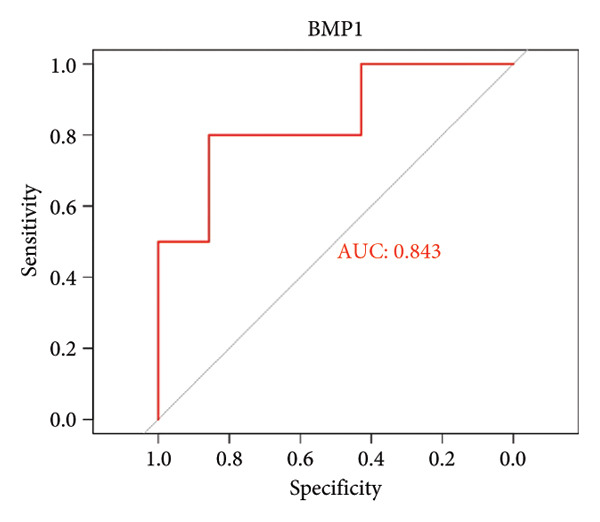
(e)

**Figure:**
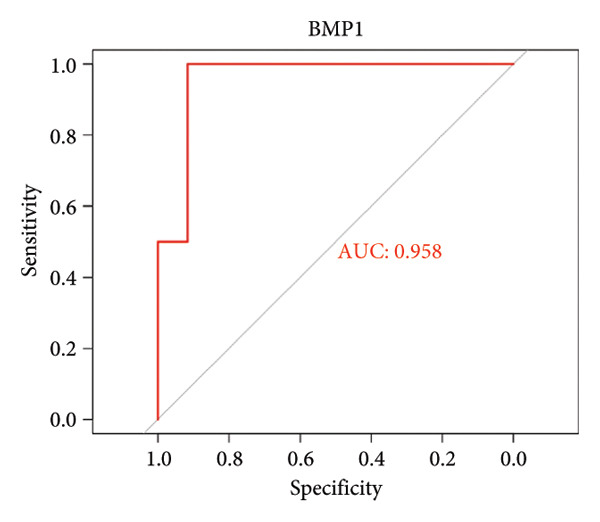
(f)

**Figure:**
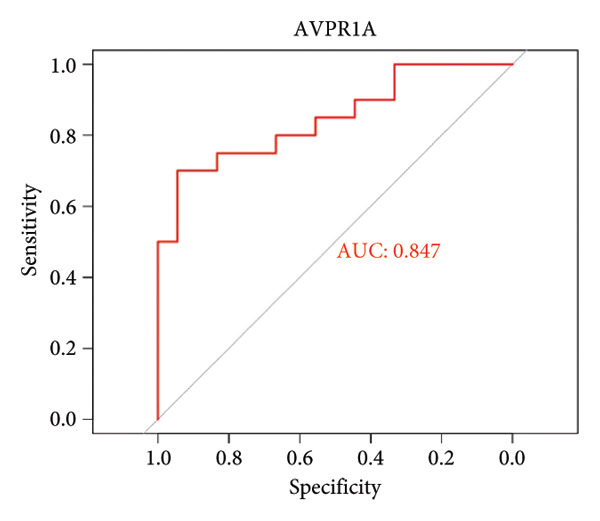
(g)

**Figure:**
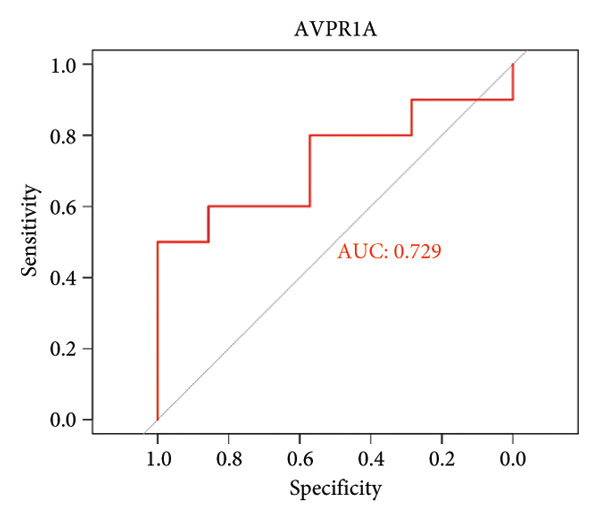
(h)

**Figure:**
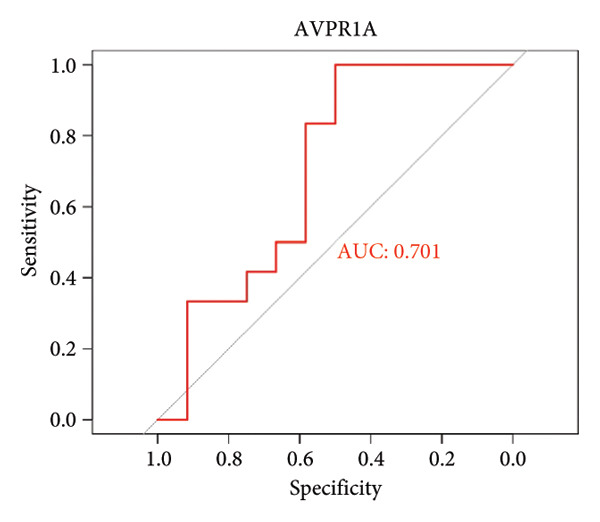
(i)

### 3.4. Functional Enrichment Analysis

To investigate the broader functional implications of our findings, we first performed ssGSEA on the GSE114007 dataset using 194 curated pathways. The ssGSEA enrichment scores were then correlated with the expression of our 26 candidate genes, and the resulting correlation coefficients were visualized in a density plot and heat map (Figures 5(a) and 5(b)). Notably, NR4A2, BMP1, and AVPR1A—which demonstrated consistent diagnostic power in multiple cohorts—also exhibited strong correlations with multiple OA‐related pathways, reinforcing their potential roles in disease pathogenesis.

Figure 5ssGSEA‐based correlation and functional enrichment analyses. (a) Density plot showing the distribution of correlation coefficients between three top candidate genes (*NR4A2*, *BMP1*, and *AVPR1A*) and the 194 curated pathway enrichment scores obtained from ssGSEA in the GSE114007 dataset. (b) Heat map depicting Pearson’s correlation coefficients between the ssGSEA scores and each of the 26 key genes across the same pathways. Positive correlations are shown in red and negative correlations in blue. (c–e) Dot plots summarizing the top 10 significantly enriched GO terms in the categories of (c) biological process, (d) cellular component, and (e) MF. (f) Dot plot of the top 10 KEGG pathways enriched in the 2246 differentially expressed genes. In each dot plot, the bubble size indicates the number of enriched genes, whereas the color indicates −log10 (*p*‐value).

**Figure:**
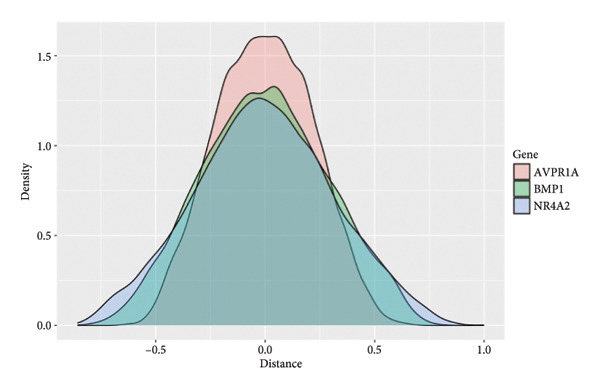
(a)

**Figure:**
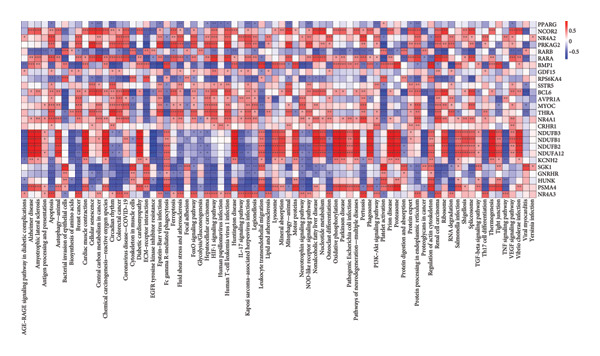
(b)

**Figure:**
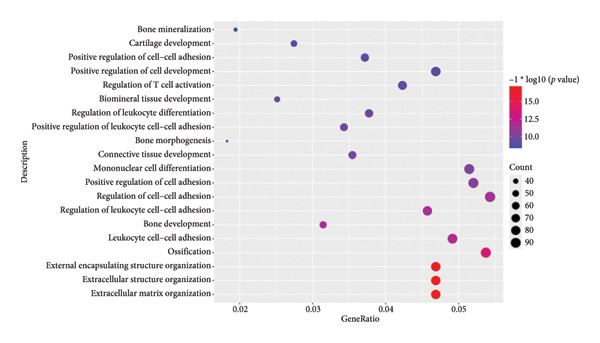
(c)

**Figure:**
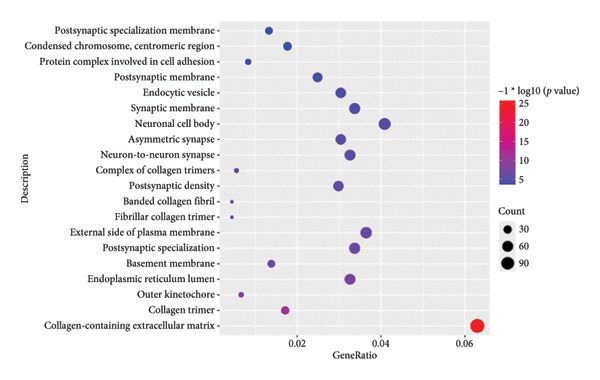
(d)

**Figure:**
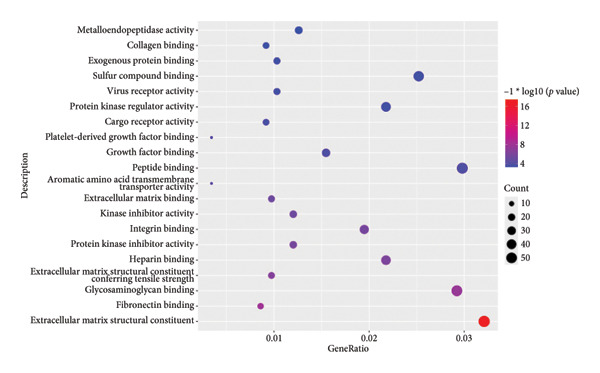
(e)

**Figure:**
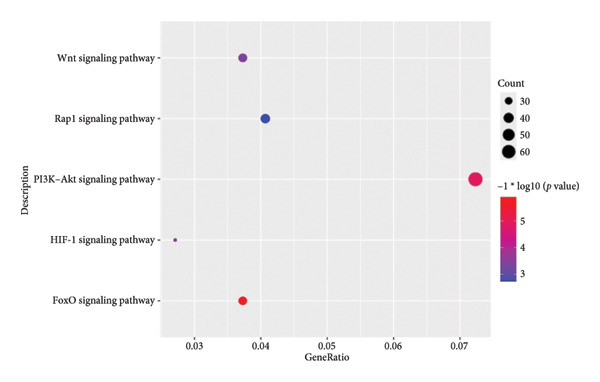
(f)

In parallel, we subjected the 2246 DEGs from GSE114007 to GO and KEGG pathway enrichment analyses (p.adjust < 0.05) via the ClusterProfiler package. The results yielded 796 significantly enriched BP terms, 33 CC terms, 24 MF terms, and 25 KEGG pathways. Dot plots of the top 10 most significant terms in each category illustrate the principal functional themes underlying OA pathobiology, including bone mineralization, cartilage development, and key signaling pathways such as Wnt, PI3K–Akt, and HIF‐1 (Figures, 5(d), 5(e), 5(f)). Together, these analyses suggest that altered extracellular matrix (ECM) homeostasis and dysregulation of multiple critical pathways may contribute to OA progression.

### 3.5. Immune Infiltration Characterization

To explore the immunological context of OA, we first conducted ssGSEA on 26 immune cell‐type gene sets in the GSE114007 dataset using the GSVA package. The resulting immune infiltration scores were then correlated with the expression levels of our 26 key OA‐associated genes via Pearson’s correlation (Figure 6(a)). Notably, multiple genes—including *NR4A2*, *BMP1*, and *AVPR1A—*exhibited strong correlations with certain T‐cell subtypes, innate immune compartments, and other immune cell populations, suggesting potential modulatory roles in OA pathogenesis.

Figure 6Correlation analysis between immune cell types, microenvironment scores, and key OA genes. Correlation of key OA‐associated genes with immune infiltration and ESTIMATE scores. (a) Heat map showing Pearson’s correlation between 26 key OA‐associated genes (rows) and ssGSEA‐derived immune infiltration scores (columns) for 26 immune cell types. (b) Heat map of Pearson’s correlation between the same 26 genes (rows) and StromalScore, ImmuneScore, ESTIMATEScore, and TumorPurity (columns) derived from the ESTIMATE algorithm. Positive correlations are in red and negative correlations in blue. Asterisks indicate statistical significance (^∗∗∗^ *p* < 0.001, ^∗∗^ *p* < 0.01, ^∗^ *p* < 0.05); “ns” represents no significance.

**Figure:**
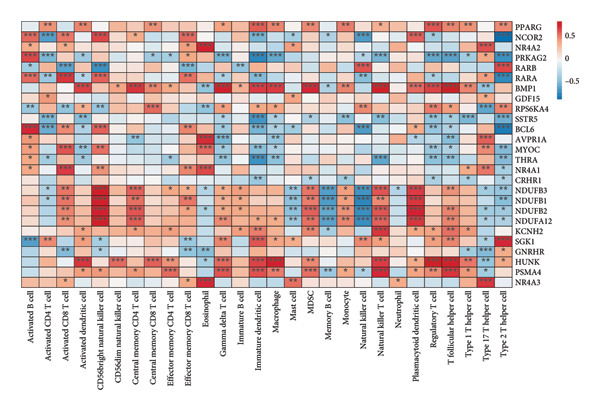
(a)

**Figure:**
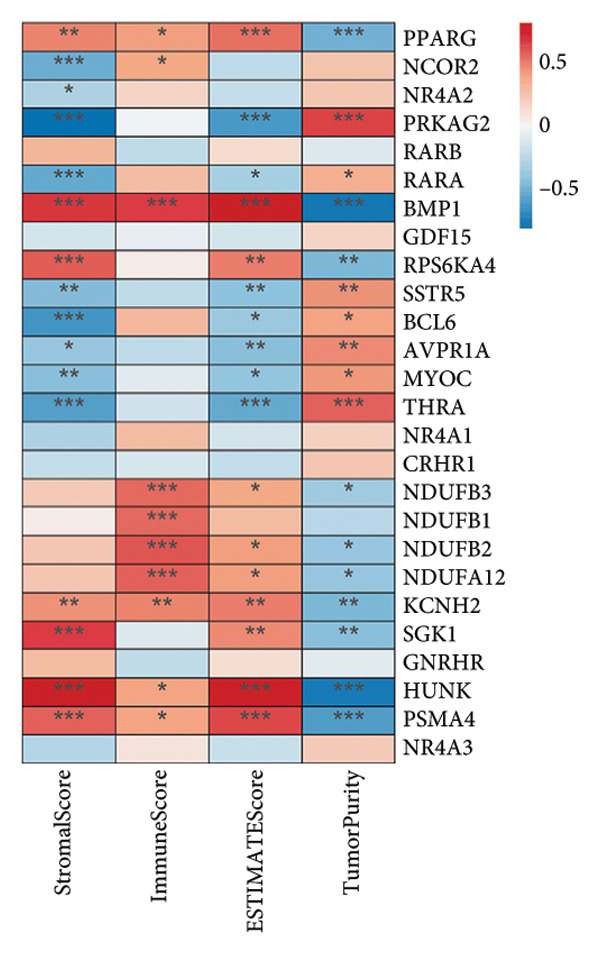
(b)

Next, we leveraged the ESTIMATE package to compute StromalScore, ImmuneScore, ESTIMATEScore, and TumorPurity values for each sample in the GSE114007 dataset. We again correlated these scores with the 26 key genes and visualized the results in a second heat map (Figure 6(b)). Consistent with the immune cell‐type data, several genes demonstrated significant correlations with the stromal and immune microenvironment scores, underscoring their potential impact on the intricate interplay between cartilage degradation and local immune infiltration in OA. These findings collectively point to an immunomodulatory dimension of OA pathology and highlight candidate genes that may serve as therapeutic or diagnostic biomarkers in the context of joint inflammation and tissue remodeling.

### 3.6. Molecular Docking of NR4A2, AVPR1A, and BMP1 With Bexarotene and Meclofenamic Acid

To further explore the therapeutic potential of our top candidate genes—NR4A2, AVPR1A, and BMP1—we selected two small‐molecule compounds, bexarotene (DB00307) and meclofenamic acid (DB00939), from ChEMBL based on their reported interactions with these gene products. The protein structures of NR4A2, AVPR1A, and BMP1 were obtained from the PDB, and molecular files for both ligands (in MOL format) were retrieved from PubChem. Molecular docking was performed using AutoDock (version 1.5.7). The docked complexes demonstrated varying binding energies and interaction residues, as summarized (Figure 7 and Table S2).

Figure 7Representative docking poses of NR4A2, AVPR1A, and BMP1 with bexarotene and meclofenamic acid. (a) Docking conformation of NR4A2 with bexarotene; (b) docking conformation of NR4A2 with meclofenamic acid; (c) docking conformation of AVPR1A with bexarotene; (d) docking conformation of AVPR1A with meclofenamic acid; (e) docking conformation of BMP1 with bexarotene; and (f) docking conformation of BMP1 with meclofenamic acid. Each panel includes an overview of the protein–ligand complex in ribbon representation (green) and a detailed view of the interacting residues (in sticks). The docked ligands are shown in red or pink, with predicted hydrogen bonds highlighted in blue.

**Figure:**
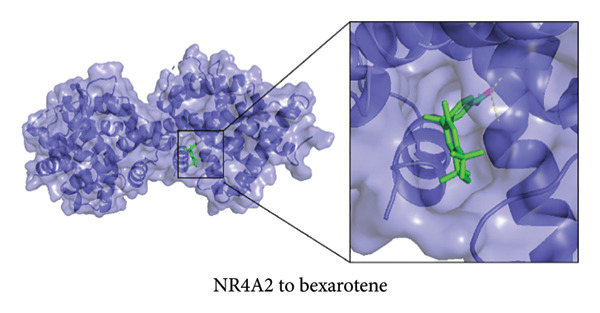
(a)

**Figure:**
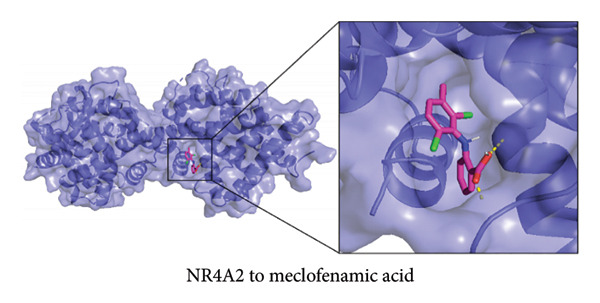
(b)

**Figure:**
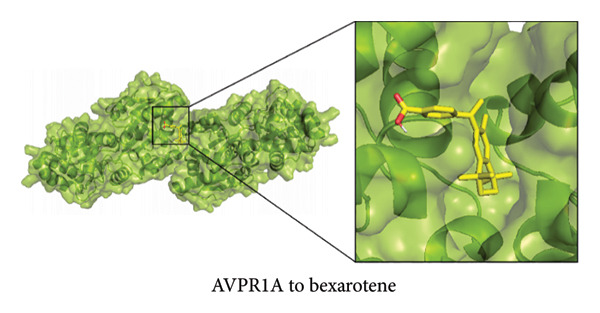
(c)

**Figure:**
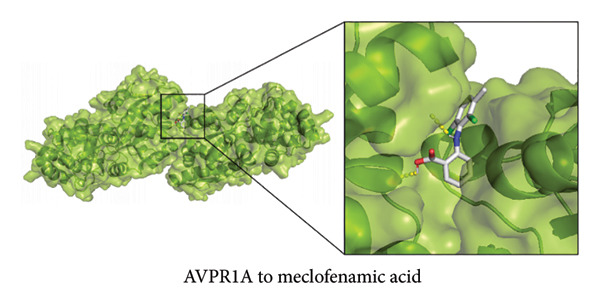
(d)

**Figure:**
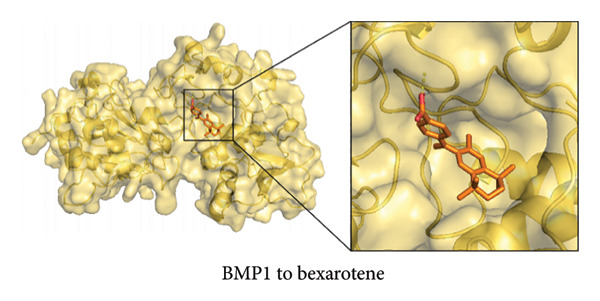
(e)

**Figure:**
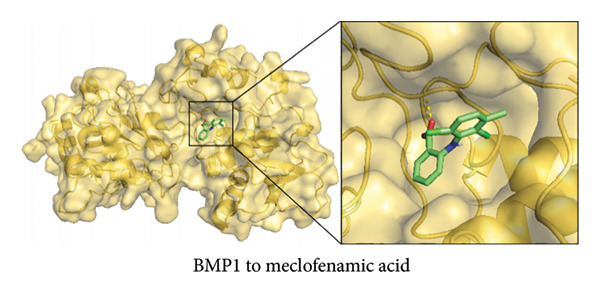
(f)

Notably, bexarotene displayed favorable binding poses and moderate binding energies with all three targets, highlighting possible allosteric or orthosteric sites for therapeutic modulation. Meclofenamic acid also formed stable complexes, albeit with slightly lower binding energies relative to bexarotene. These findings underscore the feasibility of targeting NR4A2, AVPR1A, and BMP1 through small‐molecule inhibitors or modulators. Future in vitro and in vivo studies are warranted to validate these docking predictions and to assess the therapeutic efficacy of these compounds in OA.

### 3.7. Interaction Network of Hub Genes, Enriched Pathways, and Therapeutic Drugs

**Figure 8:**
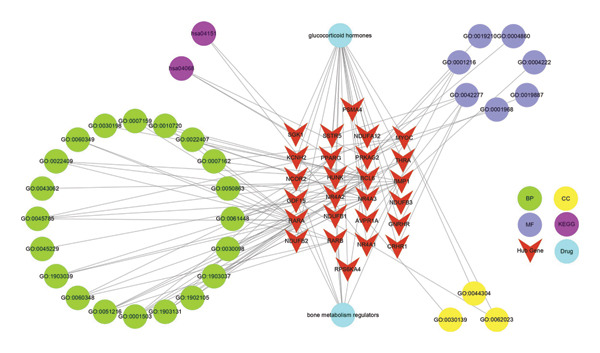
Integrated interaction network of hub genes, pathways, and drugs. Red inverted triangles represent the 26 hub genes; teal triangles represent two candidate drugs (bexarotene and meclofenamic acid); purple circles denote KEGG pathways; haze‐blue circles represent GO MF terms; yellow circles represent GO CC terms; green circles represent GO BP terms. Edges depict the interconnections between genes, pathways, and pharmacological agents, highlighting potential intervention points and mechanistic crosstalk in osteoarthritis.

To illustrate the functional and pharmacological relationships among the key pathways and putative drug targets in OA, we constructed an interaction network encompassing the 26 hub genes, the top 20 most significantly enriched GO terms (BP, MF, and CC), the top 20 enriched KEGG pathways, and two candidate small‐molecule compounds (bexarotene and meclofenamic acid). As shown in Figure 8, each red inverted triangle represents one of the 26 hub genes implicated in OA pathogenesis; the green circles denote BP terms, the haze‐blue circles indicate MF terms, the yellow circles represent CC terms, and the purple circles correspond to KEGG pathways. The two drugs identified via ChEMBL and in silico docking are shown in teal triangles. Several hub genes, including *NR4A2*, *BMP1*, and *AVPR1A*, exhibit multiple links to bone metabolism regulators, glucocorticoid hormones, and various functional pathways, reflecting the complex interplay among inflammation, ECM remodeling, and chondrocyte homeostasis in OA. This integrative network underscores potential therapeutic strategies targeting these key genes and pathways, offering a comprehensive view of the molecular architecture that drives OA progression.

### 3.8. Validation of Hub Genes in mRNA Expression Levels

**Figure 9:**
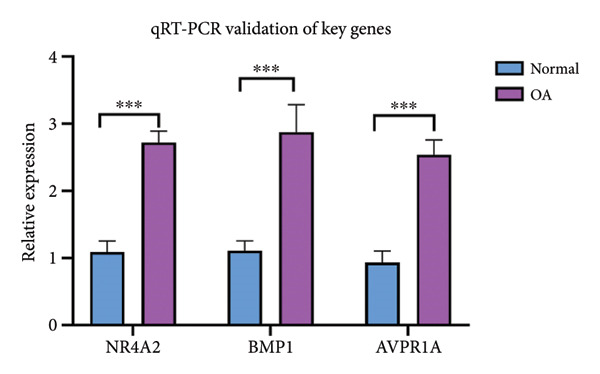
Validation of NR4A2, BMP1, and AVPR1A expression levels in osteoarthritis and normal cartilage tissues. Quantitative real‐time PCR (qRT‐PCR) was performed to assess the mRNA expression levels of NR4A2, BMP1, and AVPR1A in cartilage samples from osteoarthritis (OA) patients and healthy controls. All three genes were significantly upregulated in OA tissues compared to normal tissues (*p* < 0.001). These findings are consistent with bioinformatic predictions and support the potential of these genes as diagnostic biomarkers and therapeutic targets in OA. Data are presented as mean ± SD. Statistical analysis was performed using Welch’s *t*‐test, and *p* < 0.05 was considered statistically significant.

To validate the expression levels of key OA‐associated genes, qRT‐PCR was performed on cartilage samples obtained from OA patients and healthy controls. The results revealed that the mRNA expression levels of NR4A2, BMP1, and AVPR1A were significantly upregulated in OA cartilage tissues compared to normal tissues (*p* < 0.05). These findings suggest that these genes may contribute to OA progression by promoting inflammation, metabolic reprogramming, and endothelial dysfunction in the cartilage microenvironment. The qRT‐PCR results were consistent with the bioinformatic predictions based on the GSE114007 and GSE220243 datasets (refer to Figure 4) and were further validated at the mRNA level in clinical specimens (Figure 9). These results support the reliability of our multiomics approach and highlight the diagnostic and therapeutic potential of NR4A2, BMP1, and AVPR1A in OA.

## 4. Discussion

In this study, we applied scRNA‐seq and integrative bioinformatics to delineate the cellular and molecular underpinnings of OA. Our results revealed substantial heterogeneity within knee cartilage, identified several DEGs, and uncovered potential therapeutic targets through pathway enrichment, immune infiltration analysis, and molecular docking. These findings not only corroborate established mechanisms of cartilage degeneration but also offer new insights into disease pathogenesis and potential intervention strategies.

Our scRNA‐seq analysis uncovered eight distinct chondrocyte subpopulations in knee cartilage, with notable differences in their transcriptional profiles and relative abundances between OA and normal tissues. These findings align with previous reports suggesting that chondrocytes are not a homogenous population but instead comprise functionally discrete subtypes with varied roles in cartilage homeostasis and degeneration.

Notably, we observed significant enrichment of pathways involved in inflammation and tissue remodeling, such as the TNF, TGF‐β, and PI3K–Akt signaling pathways, in most chondrocyte clusters. These pathways have long been implicated in OA progression, where proinflammatory cytokines such as TNF‐α drive catabolic processes, whereas dysregulated TGF‐β and PI3K‐Akt signaling contribute to aberrant chondrocyte proliferation and ECM turnover. Our data add granularity by revealing that specific subpopulations of chondrocytes may preferentially engage these signaling cascades, underscoring the importance of subpopulation‐level targeting in OA therapy.

Through reanalysis of the GSE114007 bulk transcriptomic dataset, we identified 2247 DEGs, many of which reflect hallmark processes in OA—namely ECM remodeling, cartilage homeostasis, and inflammatory signaling. Notably, several collagen and matrix‐related genes (e.g., *COL1A1* and *POSTN*) were among the most upregulated in OA cartilage, consistent with the well‐established ECM dysregulation that characterizes this disease. In contrast, genes such as *HILPDA*, *ADM*, and *ATF3* were markedly downregulated, suggesting potential deficits in cellular stress responses and metabolic regulation.

By intersecting these DEGs with 205 putative OA drug targets from ChEMBL, we refined our focus to 26 key genes, including *NR4A2*, *BMP1*, and *AVPR1A*. ROC curve analyses confirmed the robust diagnostic utility of these three genes across multiple cohorts, highlighting their promise as clinically relevant biomarkers. Although BMP1 has previously been linked to cartilage and bone homeostasis, our findings extend its significance by demonstrating consistent upregulation in OA and potential associations with inflammatory signaling. Similarly, NR4A2 (a nuclear receptor) and AVPR1A (a vasopressin receptor) both have documented roles in inflammation and tissue remodeling, underscoring their plausibility as OA drivers. Its involvement in the osteogenic differentiation of mesenchymal stem cells aids in the regeneration of cartilaginous tissue, making it integral in OA studies [13]. BMP1 is a pivotal gene that regulates ECM formation. It preprocesses functional structural proteins in the ECM of active enzymes, binds with collagens I and II, and regulates the size and shape of heteromorphic fibers. The POSTN–BMP 1–LOX axis is the foundation of the mechanochemical properties of the collagen matrix [14]. Several other genes, including *POSTN*, *COL1A1*, and *COL1A2*, are closely related to collagen synthesis. The study suggests that drug development for OA should focus on intervening in the intermediate steps that promote collagen synthesis. Additionally, BMP‐1 dysregulation has been implicated in osteoporosis and OA [14].

In addition, nuclear receptor subfamily 4 group A member 2 (NR4A2) is noted for its regulatory role in inflammatory responses, which are crucial in the progression of OA. It plays a part in mediating inflammation‐related processes that may exacerbate cartilage degradation, further emphasizing its relevance to OA pathology. In a previous study, an IL‐1β‐induced OA chondrocyte model was constructed, and the results confirmed that NR4A2 was significantly upregulated in IL‐1β‐induced chondrocytes [15]. Flow cytometry analysis showed that knockdown of NR4A2 attenuated IL‐1β‐induced chondrocyte apoptosis. Silencing NR4A2 enhanced the proliferative capacity of chondrocytes with IL‐1β‐induced apoptosis. It was also studied that knockdown of NR4A2 reduced the expression of MMP3 while increasing the expression of Collagen II in IL‐1β‐induced chondrocyte apoptosis. Our findings first suggested that *NR4A2* is the key diagnostic gene in distinguishing OA samples from normal controls. Multiple studies have focused on identifying diagnostic genes related to OA. Another significant study outlined 12 hub genes, particularly *THY1*, *CTHRC1*, *SPARC*, and *COL8A1*, which were observed to be significantly upregulated in both *in vivo* and *in vitro* models when compared to controls, which can be effectively used as biomarkers to differentiate OA samples from healthy controls [16]. However, *NR4A2* is not included in this list, suggesting it may not be highlighted as a crucial differentiator in current research. Here, we found that AUC values of *NR4A2* ranged from 0.786 to 0.980 in these datasets used in this study, indicating a strong diagnostic performance of *NR4A2* in all datasets analyzed, which is consistent with the previous *in vivo* and *in vitro* findings of experiments [17].

Our data showed the negative relationship between the *NR4A2* gene and macrophages, suggesting that higher expression levels of this receptor could potentially inhibit macrophage‐mediated inflammation within the joints affected by OA [18]. This inhibition may lead to a decrease in the progression of OA symptoms, including pain and joint degeneration [19]. Understanding this interplay could pave the way for innovative therapeutic strategies aimed at enhancing NR4A2 function to mitigate inflammation in OA patients. However, an early study demonstrates that Toll‐like receptor (TLR) ligands strongly induce NR4A2, with regulation by the PI3K–Akt signaling pathway [20]. Notably, exogenous NR4A2 expression in macrophages promotes an alternative phenotype, characterized by M2 marker gene induction. Therefore, the role of *NR4A2* gene in the macrophage‐mediated inflammation needs to be further explored.

GSEA of the identified DEGs further supported the notion that OA involves multifactorial perturbations in ECM regulation, cell proliferation, and key signaling pathways such as Wnt, PI3K–Akt, and HIF‐1. These pathways collectively govern chondrocyte survival, metabolism, and matrix synthesis, and their dysregulation is consistent with progressive cartilage destruction. In parallel, our immune infiltration analyses highlighted potential interactions between chondrocytes and various immune cell types, including T‐cell subsets and innate immune cells. The observed correlations between *NR4A2*, *BMP1*, and *AVPR1A* expression and immune cell infiltration scores are particularly striking. This finding supports an emerging view of OA as a disease not solely of mechanical wear and tear but also one driven by low‐grade inflammation. Previous studies have documented immune cell infiltration in OA synovium and cartilage, but our results suggest that certain chondrocyte subpopulations may actively modulate or respond to these immune elements.

To address the translational potential of our key candidate genes, we performed molecular docking with two small‐molecule compounds—bexarotene and meclofenamic acid—predicted to target NR4A2, AVPR1A, and BMP1. Both ligands formed stable complexes with these proteins, albeit with varying binding energies and interaction residues. Although these findings are preliminary, they lay the groundwork for future experimental validation. Bexarotene showed favorable binding poses, suggesting it could modulate key molecular interactions in OA pathophysiology. Additionally, the molecular docking studies conducted reveal significant insights into the binding interactions between the AVPR1A protein and the compounds bexarotene and meclofenamic acid. Bexarotene demonstrates a stronger affinity with AVPR1A compared to meclofenamic acid, as indicated by the calculated binding energies and the nature of the interactions with the respective residues. Docking analysis indicated that bexarotene binds to NR4A2, AVPR1A, and BMP1 with binding energies in the range of −8.854 to −9.217 kcal/mol [21]. The docking analysis showed that meclofenamic acid binds to NR4A2, AVPR1A, and BMP1 with binding energies in the range of −6.536 to −7.548 kcal/mol, which are lower than those observed for bexarotene. The results underscore the differential binding affinities observed between bexarotene and meclofenamic acid, highlighting bexarotene’s potential role as a more effective ligand for AVPR1A. The stronger binding energy and multiple stabilizing interactions make bexarotene a promising candidate for further investigations in therapeutic contexts concerning AVPR1A functions and related conditions.

Despite these promising insights, certain limitations must be acknowledged. First, our scRNA‐seq dataset was derived from a relatively small number of patients and may not fully capture the interindividual variability of OA. Larger and more diverse cohorts are needed to validate the generalizability of our findings. Second, the in silico nature of our docking experiments necessitates rigorous biochemical and preclinical testing to confirm actual binding affinities and therapeutic effects. Third, although immune infiltration analyses were performed, further work is required to delineate the causal relationships between chondrocyte gene expression changes and immune cell recruitment or activation in the joint microenvironment.

Finally, although our analysis pinpointed key pathways and potential therapeutic targets, the multifactorial etiology of OA means that single‐target approaches may be insufficient to halt disease progression. Combination therapies that simultaneously address mechanical stress, inflammation, and ECM homeostasis may be necessary for robust clinical benefit.

## 5. Conclusion

In conclusion, this integrative study provides a comprehensive portrait of OA cartilage at single‐cell resolution, identifies critical molecular drivers, and suggests new directions for therapeutic development. By uniting scRNA‐seq, differential expression analysis, immune profiling, and molecular docking, we underscore the intricate interplay of chondrocyte subpopulations, inflammation, and ECM dysregulation in OA pathogenesis. Our findings highlight *NR4A2*, *BMP1*, and *AVPR1A* as high‐priority biomarkers and drug targets, offering a valuable foundation for future efforts to develop precision interventions that can ameliorate or prevent cartilage degeneration in OA.

## Ethics Statement

All data were obtained from publicly available databases. No additional ethics approval was required for the current analysis. Consent to participate is not applicable.

## Consent

The authors have nothing to report.

## Conflicts of Interest

The authors declare no conflicts of interest.

## Author Contributions

Conceptualization: Tiantian Gao, Chongshan Yang, and Yafeng Xu; data curation: Tiantian Gao, Chongshan Yang, and Ma Wan; formal analysis: Tiantian Gao, Chongshan Yang, Pingzhou Zou, Shenghui Lan, Yuan Song, and Yafeng Xu; funding acquisition: Yuan Song and Yafeng Xu; investigation: Tiantian Gao, Chongshan Yang, and Pingzhou Zou; methodology: Tiantian Gao, Yuan Song, and Yafeng Xu; project administration: Tiantian Gao and Yafeng Xu; resources: Yuan Song and Yafeng Xu; software: Yuan Song and Yafeng Xu; supervision: Tiantian Gao, Shenghui Lan, and Yuan Song; validation: Chongshan Yang, Yuan Song, and Yafeng Xu; visualization: Yikang Bi, Yuan Song, and Yafeng Xu; writing–original draft: Tiantian Gao, Chongshan Yang, Yikang Bi, and Pingzhou Zou; and writing–reviewing, and editing: Ma Wan, Shenghui Lan, Yuan Song, and Yafeng Xu.

Tiantian Gao and Chongshan Yang contributed equally to this work.

## Funding

This research was supported by the research fund of Shanghai Municipal Health Commission for Clinical Research in Medical Science (Grant No. 202040084), the National Natural Science Foundation of China (Grant No. 81601902), the Medical Research Projects of Shanghai Eighth People’s Hospital (Grant No. SHBY202501), the Key Project of the Seed Program of Shanghai Municipal Health Commission for Medical Innovation and Transformation (Grant No. 2025ZZ1009), the Oriental Talents Youth Program of Shanghai (to Shenghui Lan), and the Key Cultivation Project of the Xuhui District Medical Scientific Research Program (Grant No. SHXH202509).

## Supporting Information

The supporting information provides additional figures and tables supporting the single‐cell and bioinformatic analyses. Figure S1 presents QC metrics and NR4A2 expression across chondrocyte subsets. Tables S1–S3 summarize the diagnostic performance of key OA‐related genes, molecular docking results, and primer sequences used for qRT‐PCR validation. These materials enhance the robustness and reproducibility of the study.

## Supporting information


**Supporting Information** Additional supporting information can be found online in the Supporting Information section.

## Data Availability

The data that support the findings of this study are available from the corresponding author upon reasonable request.

## References

[1] Bijlsma J. W. , Berenbaum F. , and &Lafeber F. P. , Osteoarthritis: An Update With Relevance for Clinical Practice, Lancet. (2011) 377, no. 9783, 2115–2126, 10.1016/s0140-6736(11)60243-2, 2-s2.0-79959341732. 21684382

[2] Chen Y. , Zhang Y. , Ge Y. , and &Ren H. , Integrated Single-Cell and Bulk RNA Sequencing Analysis Identified Pyroptosis-Related Signature for Diagnosis and Prognosis in Osteoarthritis, Scientific Reports. (2023) 13, no. 1, 10.1038/s41598-023-44724-0. PMC10584952 37853066

[3] Longo S. K. , Guo M. G. , Ji A. L. , and &Khavari P. A. , Integrating Single-Cell and Spatial Transcriptomics to Elucidate Intercellular Tissue Dynamics, Nature Reviews Genetics. (2021) 22, no. 10, 627–644, 10.1038/s41576-021-00370-8. PMC9888017 34145435

[4] Ramachandran P. , Matchett K. P. , Dobie R. , Wilson-Kanamori J. R. , and &Henderson N. C. , Single-Cell Technologies in Hepatology: New Insights Into Liver Biology and Disease Pathogenesis, Nature Reviews Gastroenterology & Hepatology. (2020) 17, no. 8, 457–472, 10.1038/s41575-020-0304-X. 32483353

[5] Ghorbaninejad M. , Farahi S. , Mirzaeian F. , Khodabandehloo F. , Hosseini S. , and &Baghaban Eslaminejad M. , Single-Cell Analysis Approaches in Cartilage Diseases Diagnosis and Therapies, Cartilage: From Biology to Biofabrication. (2023) Springer, 67–95.

[6] Hu X. , Li Z. , Ji M. , Lin Y. , Chen Y. , and &Lu J. , Identification of Cellular Heterogeneity and Immunogenicity of Chondrocytes via Single-Cell RNA Sequencing Technique in Human Osteoarthritis, Frontiers in Pharmacology. (2022) 13, 10.3389/fphar.2022.1004766. PMC9562112 36249797

[7] Nedunchezhiyan U. , Varughese I. , Sun A. R. , Wu X. , Crawford R. , and &Prasadam I. , Obesity, Inflammation, and Immune System in Osteoarthritis, Frontiers in Immunology. (2022) 13, 10.3389/fimmu.2022.907750. PMC9289681 35860250

[8] Sanchez-Lopez E. , Coras R. , Torres A. , Lane N. E. , and &Guma M. , Synovial Inflammation in Osteoarthritis Progression, Nature Reviews Rheumatology. (2022) 18, no. 5, 258–275, 10.1038/s41584-022-00749-9. 35165404 PMC9050956

[9] Woodell‐May J. E. and Sommerfeld S. D. , Role of Inflammation and the Immune System in the Progression of Osteoarthritis, Journal of Orthopaedic Research. (2020) 38, no. 2, 253–257, 10.1002/jor.24457, 2-s2.0-85073785492. 31469192

[10] Edgar R. , Domrachev M. , and &Lash A. E. , Gene Expression Omnibus: NCBI Gene Expression and Hybridization Array Data Repository, Nucleic Acids Research. (2002) 30, no. 1, 207–210, 10.1093/nar/30.1.207. 11752295 PMC99122

[11] Gaulton A. , Hersey A. , Nowotka M. et al., The ChEMBL Database in 2017, Nucleic Acids Research. (2017) 45, no. D1, D945–D954, 10.1093/nar/gkw1074, 2-s2.0-85016137105. 27899562 PMC5210557

[12] Li R. , Li Y. , Liang X. , Yang L. , Su M. , and &Lai K. P. , Network Pharmacology and Bioinformatics Analyses Identify Intersection Genes of Niacin and CoviD-19 as Potential Therapeutic Targets, Briefings in Bioinformatics. (2021) 22, no. 2, 1279–1290, 10.1093/bib/bbaa300. 33169132 PMC7717147

[13] Aref-Eshghi E. , Understanding the Role of Transforming Growth Factor Beta Signalling and Epigenomics in Osteoarthritis, 2016, Memorial University of Newfoundland.

[14] Weng P.-W. , Pikatan N. W. , Setiawan S. A. et al., Role of GDF15/MAPK14 Axis in Chondrocyte Senescence as a Novel Senomorphic Agent in Osteoarthritis, International Journal of Molecular Sciences. (2022) 23, no. 13, 10.3390/ijms23137043. PMC9266723 35806043

[15] Liu W. , Chen Y. , Zeng G. et al., Single-Cell Profiles of age-Related Osteoarthritis Uncover Underlying Heterogeneity Associated With Disease Progression, Frontiers in Molecular Biosciences. (2022) 8, 10.3389/fmolb.2021.748360. PMC8784753 35083277

[16] Wen Y. , Zou M. , and &Chen C. , Diagnostic Biomarkers in Knee Osteoarthritis: Based on Bioinformatics and Experimental Verification in Vivo and in Vitro, Journal of Orthopaedic Surgery. (2024) 32, no. 2, 10.1177/10225536241267027. 39110784

[17] Lilley C. M. , Alarcon A. , Ngo M.-H. , Araujo J. S. , Marrero L. , and &Mix K. S. , Orphan Nuclear Receptor NR4A2 is Constitutively Expressed in Cartilage and Upregulated in Inflamed Synovium From hTNF-Alpha Transgenic Mice, Frontiers in Pharmacology. (2022) 13, 10.3389/fphar.2022.835697. PMC9067626 35529439

[18] Tarricone E. , Mattiuzzo E. , Belluzzi E. et al., Anti-Inflammatory Performance of Lactose-Modified Chitosan and Hyaluronic Acid Mixtures in an in Vitro Macrophage-Mediated Inflammation Osteoarthritis Model, Cells. (2020) 9, no. 6, 10.3390/cells9061328. PMC7349682 32466461

[19] Xie J. , Huang Z. , Yu X. , Zhou L. , and &Pei F. , Clinical Implications of Macrophage Dysfunction in the Development of Osteoarthritis of the Knee, Cytokine & Growth Factor Reviews. (2019) 46, 36–44, 10.1016/j.cytogfr.2019.03.004, 2-s2.0-85063291346. 30910350

[20] Mahajan S. , Saini A. , Chandra V. et al., Nuclear Receptor Nr4a2 Promotes Alternative Polarization of Macrophages and Confers Protection in Sepsis, Journal of Biological Chemistry. (2015) 290, no. 30, 18304–18314, 10.1074/jbc.m115.638064, 2-s2.0-84937851519. 25953901 PMC4513091

[21] Windshugel B. , Structural Insights Into Ligand-Binding Pocket Formation in Nurr1 by Molecular Dynamics Simulations, Journal of Biomolecular Structure and Dynamics. (2019) 37, no. 17, 4651–4657, 10.1080/07391102.2018.1559099, 2-s2.0-85060927719. 30582418

