# Supplementary material for: Single‐Cell Transcriptomics and Integrated Bioinformatic Analysis Reveal Critical Biomarkers and Immune Infiltration Characteristics in Osteoarthritis
Source: Genet Res (Camb). 2026 Jan 6;2026:1174568. doi: 10.1155/genr/1174568 (PMC12771611; doi:10.1155/genr/1174568)
Supplement: Supplementary file 1 — Supporting Information Additional supporting information can be found online in the Supporting Information section. [file GENR-2026-1174568-s001.docx]

**Supplementary figures**

**Figure S1 Quality control metrics for scRNA‐seq data (GSE220243).** (A) Scatter plots illustrating the relationships among total unique molecular identifiers (nCount_RNA), detected gene features (nFeature_RNA), and mitochondrial RNA content (percent.mt) per cell, colored by sample identity (GSM identifiers). (B) Violin plots showing the distribution of nFeature_RNA, nCount_RNA, percent.mt, and percent.ribo across samples, demonstrating consistent QC metrics before filtering. (C) Principal component analysis (PCA) of high‐quality cells. Each color represents an individual sample. (D) Elbow plot of the standard deviations of the top 20 principal components (PCs), guiding the choice of PCs for downstream clustering. (E) Violin plot of selected candidate gene expression (*NR4A2*) across chondrocyte subpopulations, highlighting differential expression among the eight clusters.

**Supplementary tables**

**Table S1 The AUC values (>0.7) of 26 key genes in the three datasets**

| **Gene names** | **AUC values** |
| --- | --- |
| **GSE114007** |  |
| PPARG | 0.761 |
| NCOR2 | 0.725 |
| NR4A2 | 0.831 |
| PRKAG2 | 0.881 |
| RARB | 0.725 |
| RARA | 0.9 |
| BMP1 | 0.95 |
| GDF15 | 0.758 |
| RPS6KA4 | 0.853 |
| SSTR5 | 0.744 |
| BCL6 | 0.836 |
| AVPR1A | 0.847 |
| MYOC | 0.878 |
| THRA | 0.942 |
| NR4A1 | 0.814 |
| NDUFB2 | 0.725 |
| NDUFA12 | 0.717 |
| KCNH2 | 0.797 |
| SGK1 | 0.864 |
| GNRHR | 0.822 |
| HUNK | 0.9 |
| PSMA4 | 0.856 |
| NR4A3 | 0.856 |
| **GSE82107** | |
| NR4A2 | 0.786 |
| PRKAG2 | 0.829 |
| BMP1 | 0.843 |
| BCL6 | 0.743 |
| AVPR1A | 0.729 |
| MYOC | 0.771 |
| NDUFB1 | 0.8 |
| NDUFA12 | 0.857 |
| SGK1 | 0.814 |
| **GSE98918** | |
| PPARG | 0.889 |
| NR4A2 | 0.952 |
| BMP1 | 0.958 |
| SSTR5 | 0.722 |
| AVPR1A | 0.701 |
| THRA | 0.833 |
| NR4A1 | 0.931 |
| PSMA4 | 0.729 |

**Table S2 Summary of Docking Results for NR4A2, AVPR1A, and BMP1 with Bexarotene and Meclofenamic Acid**

| Gene names | Compound | Binding energy |
| --- | --- | --- |
| NR4A2 | DB00307 | -8.854 kcal/mol |
|  | DB00939 | -6.536 kcal/mol |
| AVPR1A | DB00307 | -9.217 kcal/mol |
|  | DB00939 | -7.548 kcal/mol |
| BMP1 | DB00307 | -8.986 kcal/mol |
|  | DB00939 | -7.503 kcal/mol |

Note: Negative binding energy values represent a thermodynamically favorable interaction, and the indicated residues reflect primary predicted contact sites.

**Table S3: The primer used for three gene.**

| Targeted gene | Forward (5′-3′) | Reverse (3′-5′) |
| --- | --- | --- |
| NR4A2 | GGTCAAAGAAGTGGTTCGC | CACTGATCAGACTCACCGG |
| AVPR1A | TGACGGCTTACATCGTCTG | TTTCCGATTCGGTCCAGAC |
| BMP1 | AACTTCACTGGTAGCCAGAG | CTCCAGGAAGGTGACACAG |
| GAPDH | TCAAGATCATCAGCAATGCC | CGATACCAAAGTTGTCATGGA |
